# Anticancer properties and enhancement of therapeutic potential of cisplatin by leaf extract of *Zanthoxylum armatum* DC.

**DOI:** 10.1186/s40659-015-0037-4

**Published:** 2015-08-20

**Authors:** Thangjam Davis Singh, Heikrujam Thoihen Meitei, Adhikarimayum Lakhikumar Sharma, Asem Robinson, Lisam Shanjukumar Singh, Thiyam Ramsing Singh

**Affiliations:** Department of Biotechnology, Manipur University, Imphal, 795003 India

**Keywords:** *Zanthoxylum armatum*, Apoptosis, ERK activation, Cisplatin sensitization, DNA damage

## Abstract

**Background:**

Clinical use of chemotherapeutic drug, cisplatin is limited by its toxicity and drug resistance. Therefore, efforts continue for the discovery of novel combination therapies with cisplatin, to increase efficacy and reduce its toxicity. Here, we screened 16 medicinal plant extracts from Northeast part of India and found that leaf extract of *Zanthoxylumarmatum* DC. (ZALE) induced cytotoxicity as well as an effect on the increasing of the efficiency of chemotherapeutic drugs (cisplatin, mitomycin C and camptothecin). This work shows detail molecular mechanism of anti-cancer activity of ZALE and its potential for combined treatment regimens to enhance the apoptotic response of chemotherapeutic drugs.

**Results:**

ZALE induced cytotoxicity, nuclear blebbing and DNA fragmentation in HeLA cells suggesting apoptosis induction in human cervical cell line. However, the apoptosis induced was independent of caspase 3 activation and poly ADP ribose polymerase (PARP) cleavage. Further, ZALE activated Mitogen-activated protein kinases (MAPK) pathway as revealed by increased phosphorylation of extracellular-signal-regulated kinases (ERK), p38 and c-Jun N-terminal kinase (JNK). Inhibition of ERK activation but not p38 or JNK completely blocked the ZALE induced apoptosis suggesting an ERK dependent apoptosis. Moreover, ZALE generated DNA double strand breaks as suggested by the induction γH2AX foci formation. Interestingly, pretreatment of certain cancer cell lines with ZALE, sensitized the cancer cells to cisplatin and other chemotherapeutic drugs. Enhanced caspase activation was observed in the synergistic interaction among chemotherapeutic drugs and ZALE.

**Conclusion:**

Purification and identification of the bio-active molecules from the ZALE or as a complementary treatment for a sequential treatment of ZALE with chemotherapeutic drugs might be a new challenger to open a new therapeutic window for the novel anti-cancer treatment.

**Electronic supplementary material:**

The online version of this article (doi:10.1186/s40659-015-0037-4) contains supplementary material, which is available to authorized users.

## Background

Despite early promising results, cancer treatment with modulators has not improved due to the emergence of drug resistance. This may be explained by the fact that there are alternate resistance mechanisms, controlled by different families of genes, such as those involved in apoptosis. Therefore, there is a need to develop new anticancer drugs and novel regimens that are capable of killing drug-resistant cancer cells. Understanding that the molecular mechanisms of chemo-resistance implicate several ways and genes, including genes associated with apoptosis, the studies of new chemotherapeutic drugs or new protocols for cancer treatment are important and needed.

Apoptosis is a mode of programmed cell death that is used by multicellular organisms to remove cell detachment, cell shrinkage, chromatin condensation, DNA degradation, and plasma membrane blebbing [1]. Apoptosis may be caspase-dependent, or caspase-independent pathways [2]. Regulation of apoptosis is of paramount importance in cancer biology because a large numbers of cancer cells are defective in the regulation of apoptosis [3]. Therefore, finding of new biologically active molecules that can activate the highly regulated apoptotic cell death in cancer cells could be an important issue to improve the cancer treatment.

Natural products have played an important role as sources of effective anti-cancer agents. More than 60 % of drugs for cancer treatment are of natural origin, particularly derived from plants [4]. Additionally, secondary metabolites of plants are considered to be important sources of molecules with a great potential for chemotherapy [5]. Research in plants, therefore, represents an invaluable source for discovering of new substances and secondary metabolites. From approximately more than 300,000 plant species reported, only a small percentage has been the subject of phytochemical and biological activity studies [6]. Here we screen the medicinal plants from north eastern part of India and found that leaf extracts of *Zanthoxylum armatum* DC. (local name: Mukthrubi) induced apoptosis and also sensitized the cancer cells to chemotherapeutic drugs.

*Zanthoxylum armatum* DC. is an aromatic medicinal plant in the Rutaceae family, and the plant parts like leaves, stem, bark, fruits, seeds and roots possess medicinal properties and are used in indigenous medicine preparation against various diseases like asthma, bronchitis, indigestion, varicose veins, diarrhea, rheumatism, dyspepsia, cholera and toothache. The different extracts from the plant have different pharmacological activities such as antioxidative [7], anti-inflammatory [8, 9], antimicrobial, insecticidal, larvicidal [10, 11], piscicidal [12], hepatoprotective [13], antitumor [14], and immunomodulation activity [15]. Aqueous extract of *Z. armatum* induced cellular and nuclear damaged coupled with inhibition of mitotic activity in plant [16].

The current study was undertaken to evaluate the cytotoxic and genotoxic potential of the crude methanol extract of *Z. armatum* leaves (ZALE) in human cervical cell line (HeLa) and to gain insight into the molecular mechanism(s) by which the extract exert the cytotoxicity and chemo sensitize the cancer cells.

## Results

### ZALE induced apoptosis in human cervical cancer cell line

**Fig. 1:**
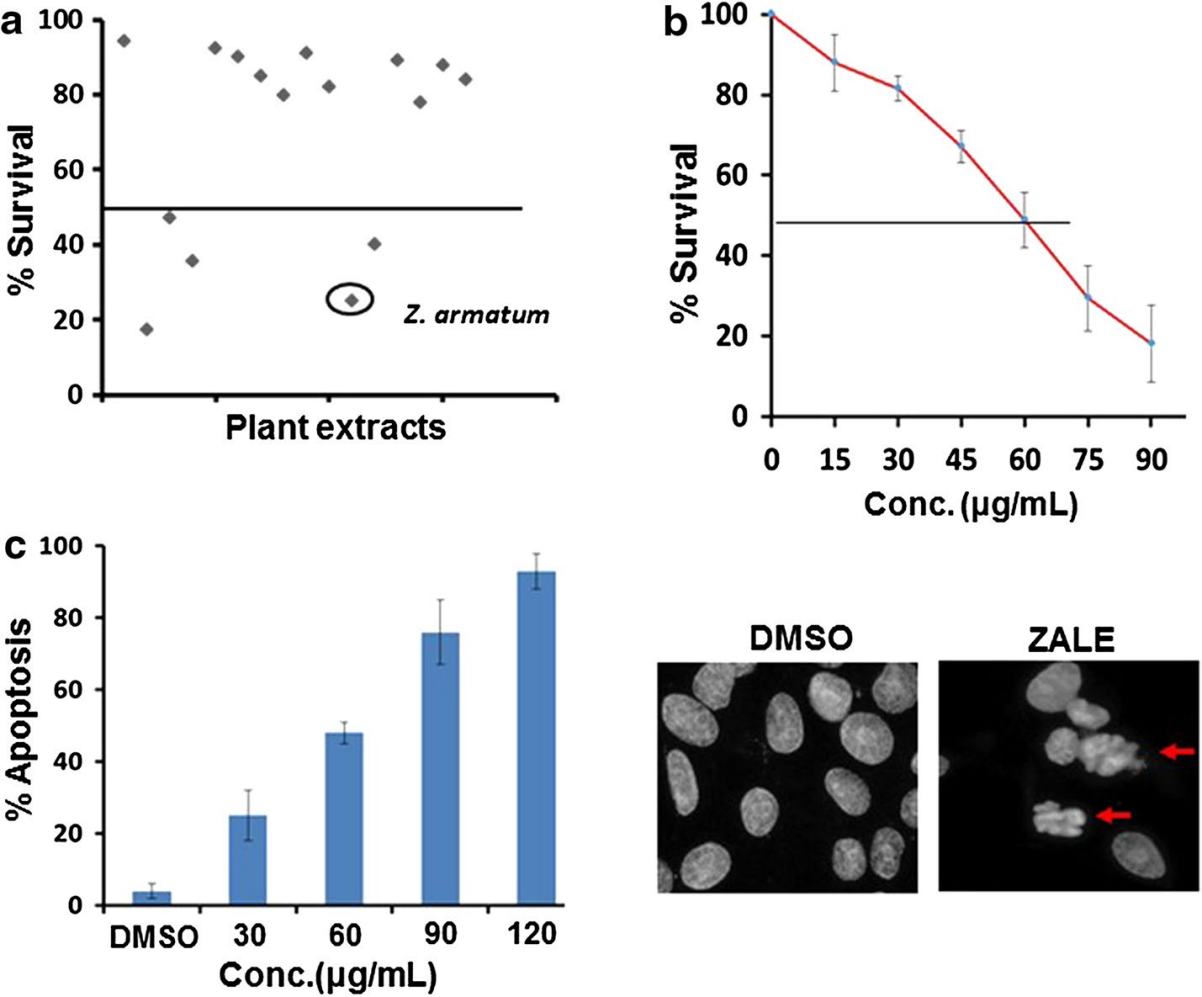
Screening of apoptosis inducing plant extracts. **a** *Graph s*howing efficacy of plant extracts screened for cytotoxicity. HeLa cells were treated with plant extracts (80 μg/ml) and the viable cells were measured with the Cell Titer 96 Proliferation Assay (Promega). **b** *Graph* showing the dose depedent inhibton of cell proliferation. Cells were treated either with DMSO or with the indicated concentration of ZALE and the viable cells were measured as above. The data represent the percentage growth compared with DMSO. **c** *Left panel* quantification of apoptotic nuclei observed in HeLa cells treated with either DMSO or ZALE with the indicted extract concentration for 48 h. *Right panel* representative DAPI stained images. *Arrows* indicate apoptotic fragmented nuclei

To investigate the plants which induced cytotoxicity, 16 medicinal plants from the Manipur, a Northeast part of India were screened. Five extracts, including ZALE show IC_50_ less than 80 μg/ml while the remaining 11 extracts show IC_50_ more than 80 μg/ml (Fig. 1a). In this paper we have selected ZALE for further study. Treatment of HeLa cells with 80 μg/ml of ZALE for 48 h showed marked morphological changes and cytotoxicity in dose dependent manner. Most of the cells were rounded up and detached from the tissue culture dish (Additional file 1: Figure S1). Determination of the number of viable cells in proliferation or cytotoxicity assays showed a dose dependent inhibition of cell proliferation of HeLa cells and the IC_50_ of the ZALE was approximately 60 μg/ml (Fig. 1b).

Necrosis and apoptosis are two well-described pathways of cell death. The end stages of apoptosis are often characterized by the appearance of membrane blebbing, the appearance of highly condensed chromatin and activation of an endonucleolytic process, that leads to the cleavage of chromosomal DNA into oligosomal DNA that can be observed as a distinct laddering pattern on an ethidium bromide-stained agarose gel. To determine whether the cytotoxicity observed was due to apoptosis, the cells were stained with 4, 6-diamidino-2-phenylindole (DAPI) and observed under microscope. Similar to cell cytotoxicity, increased micronuclei and nuclear ablation were also observed in the cells treated with ZALE (Fig. 1c). To further confirm that ZALE treatment induced apoptosis, nucleosomal DNA fragmentation and single cell comet assay were analyzed. Fluorescent comets with varying length of “tails” and increased nucleosomal DNA fragmentations were visible when HeLa cells were treated with different concentration of ZALE (Additional file 1: Figure S2 and data not shown). Taken together, these data clearly suggest that ZALE induced apoptosis in HeLa cell line.

### ZALE induced apoptosis is independent of caspase 3 activation and PARP cleavage

**Fig. 2:**
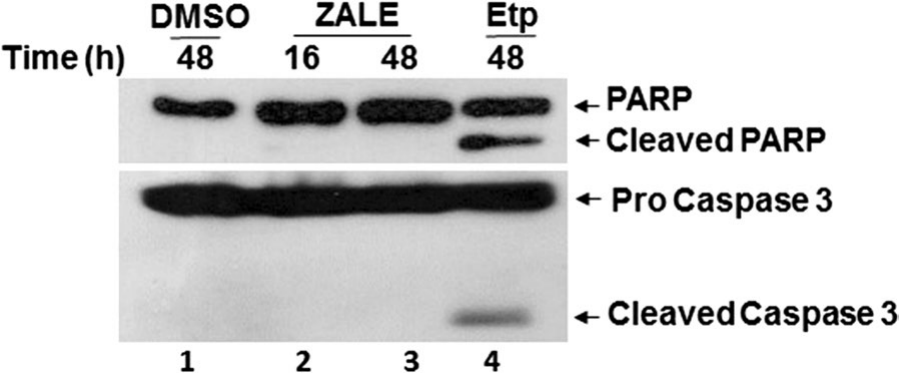
ZALE failed to activate caspase 3. *Immuoblot for the analysis of caspase3 activtion and PARP cleavage.* HeLa cells were treated with DMSO (negative control), ZALE (60 μg/ml) or etoposide (Etp) for the indicated time. Total cell lysates were immunoblotted with the indicated antibodies. Etp treated sample was included as a positive control

Apoptosis can be classified into caspase-dependent or caspase-independent pathways. All caspases are synthesized in cells as catalytically inactive zymogens and are activated by proteolytic cleavage. Activated caspases cleaved key cellular components that are required for normal cellular functions, including structural proteins in the cytoskeleton and nuclear proteins, such as DNA repair enzymes, including the enzyme PARP [17]. The cleavage of PARP by caspase is often used as a marker for caspase activity. To delineate the molecular mechanism of apoptosis induced by ZALE, we analyzed the caspase 3 activation and PARP cleavage by immunoblotting. As expected, the etoposide treated positive control cells show caspase 3 activation and PARP cleavage (Fig. 2, lane 4). However, ZALE treated cells did not show any caspase 3 activation or PARP cleavage (Fig. 2). Similar results were observed when the cells were treated at shorter time points of 8, 16 and 24 h (Fig. 2, lane 2 and data not shown), suggesting for apoptosis without activating caspase 3 and PARP cleavage.

### ZALE activates MAPK pathway

**Fig. 3:**
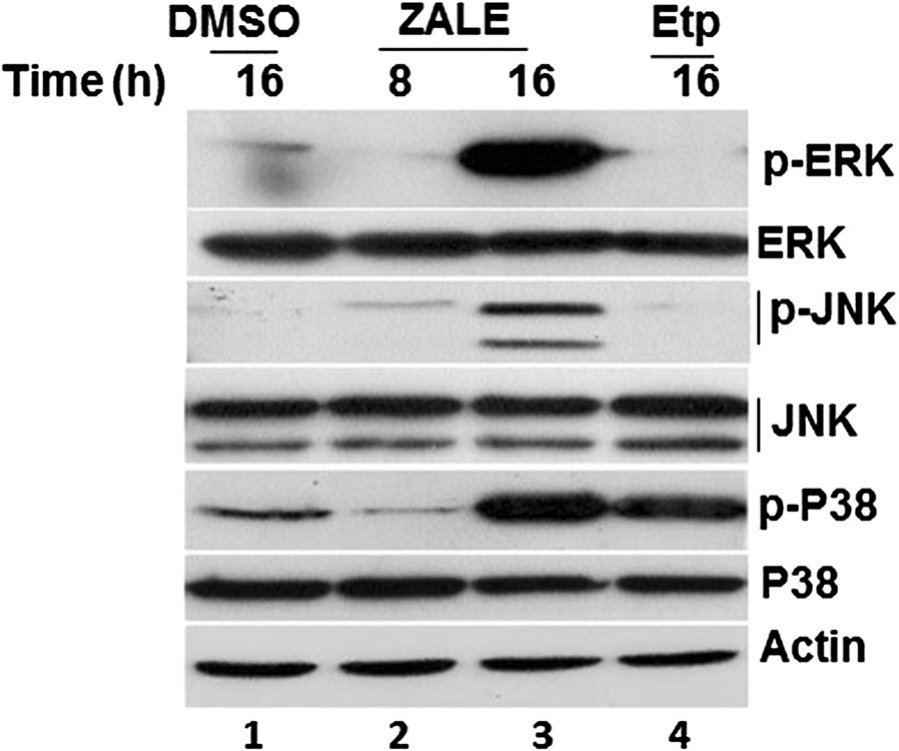
Analysis of MAPK pathway activation by ZALE. Immuoblot showing MAPK pathway activtion. HeLa cells were treated with ZALE (60 μg/ml) for 8 and 16 h and total cell lysates were immunoblotted with the indicated MAPK pathway antibodies. DMSO and Etoposide (Etp) treated samples were included as negative and positive

Recent literatures have shown MAPK pathway dependent apoptosis without activating caspase 3 or PARP cleavage [18]. This prompted us to investigate if ZALE treated cells also activated MAPK pathways and induced apoptosis independent of caspase 3 activation and PARP cleavage. Immunoblot analysis of MAPK pathways activation using phosphorylated forms of JNK, ERK and p38 show that none of the MAPK pathways were activated in 8 h; however all the MAPK pathways were activated in 16 h as determined by increased phosphorylation of ERK, JNK and p38 (Fig. 3). Maximum phosphorylation was found using anti-pERK (Fig. 3, Lane 3). Interestingly, ZALE also activated p38 similar to the activation by chemotherapeutic drug, etoposide.

### Roles of MAPK pathway in ZALE induced apoptosis

**Fig. 4:**
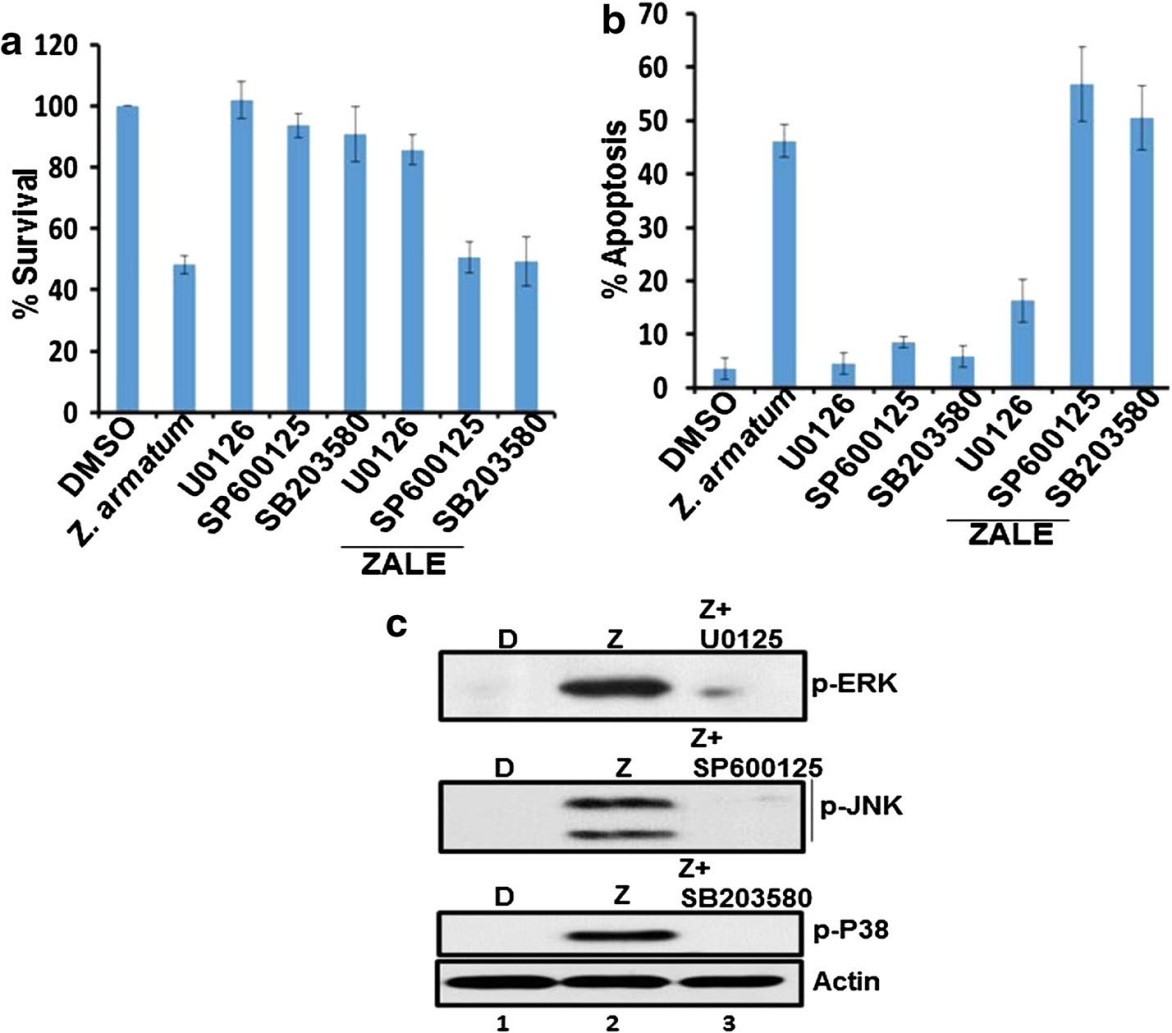
ZALE induced ERK dependent apoptosis. **a** ZALE induced cytotoxicity to HeLa cells is ERK dependent. HeLa cells were treated with DMSO, ZALE, U0126 (ERK inhibitor), SP600125 (JNK inhibitor), or combination of inhibitor and ZALE. For combination treatment, cells were pretreated with the inhibitors 2 h prior to the treatment with ZALE (60 μg/ml). After 48 h viable cells were measured. The data represent the percentage growth compared with DMSO treated cells and show 1 representative result of 3 independent experiments with standard deviations. **b** Quantification of apoptotic nuclei observed after DAPI staining in the HeLa cells treated with either DMSO or ZALE or the MAPK inhibitors-ZALE combination for 48 h. **c** Immunblot showing the inhibition of activation of ERK, p38 and JNK by their respective inhibitors. Cells were treated with D (DMSO), Z (ZALE) or Z and the specific inhibitors

We have shown that ZALE activated MAPK pathways. Further, we wanted to investigate whether the ZALE induced apoptosis is due to the activation of MAPK pathways. Interestingly, the MEK1/2 inhibitor U0126, which inhibits MEK1/2 activity and thereby blocks ERK1/2 phosphorylation, completely abrogated the ZALE induced apoptosis and cell cytotoxicity. Cell were rescued from the anti-proliferative activity as well as cell death (Fig. 4a, b) suggesting that phosphorylated ERK1/2 is important for ZALE induced apoptosis. The cell death was not inhibited by the JNK inhibitor, SP600125 and the p38 inhibitor, SB203580 (Fig. 4a, b). Inhibitions of ERK, JNK and p38 by their respective inhibitors, U0126, SP600125, SB203580 were confirmed by immunoblotting (Fig. 4c).

### ZALE induced DNA damage

**Fig. 5:**
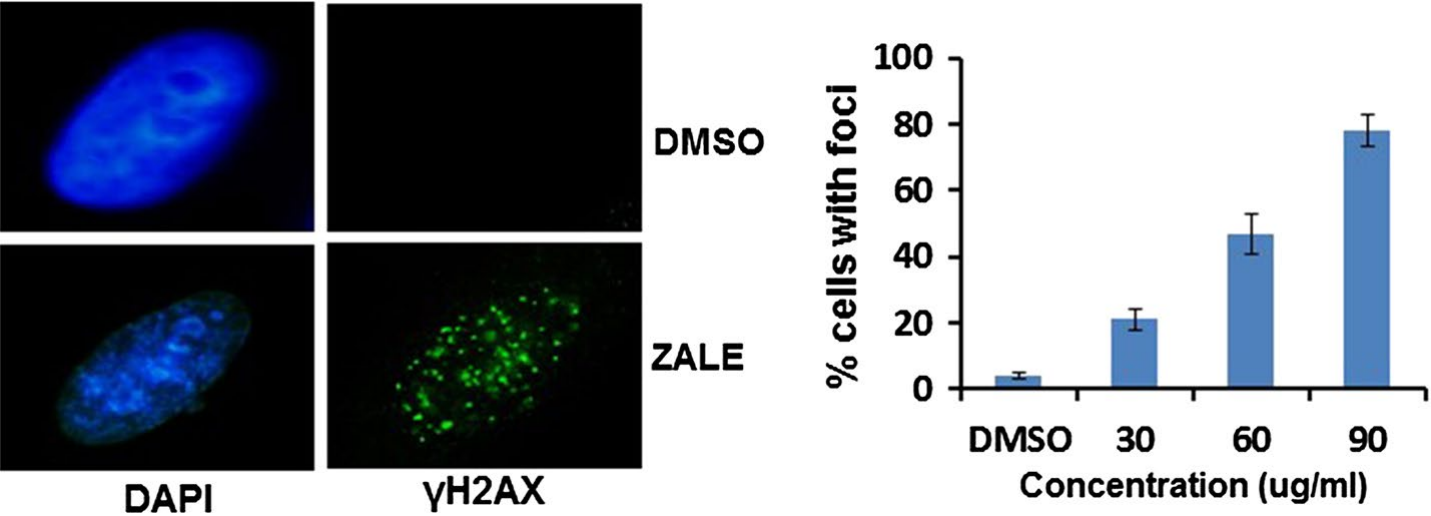
ZALE induced γH2AX foci formation. *Left panel* representative images showing γH2AX foci in HeLa cells treated with ZALE or the DMSO only. *Right panel* quantification of the number of cells with more than 5 foci in the cells treated with different doses of ZALE as indicated or DMSO treated control. HeLa cells were treated with the indicated concentration of ZALE for 24 h and the cells were fixed and the γH2AX foci were analyzed by immunofluorescent using anti-γH2AX antibody

Histone H2AX was phosphorylated in response to DNA damages such as double strand breaks by γ-irradiation. Immuno-fluorescence analysis for γH2AX foci formation, observed an increased in γH2AX foci (more than five foci per cell) in ZALE treated HeLa cells in a dose dependent manner (Fig. 5) indicating generation of DNA double strand breaks. Interestingly, the amount of cell death was correlated with the number of the cells with γH2AX foci. When the cells were treated with a dose of 60 μg/ml (IC_50_) of ZALE, half of the cells show γH2AX foci.

### Synergistic interaction between ZALE and chemotherapeutic drugs

**Fig. 6:**
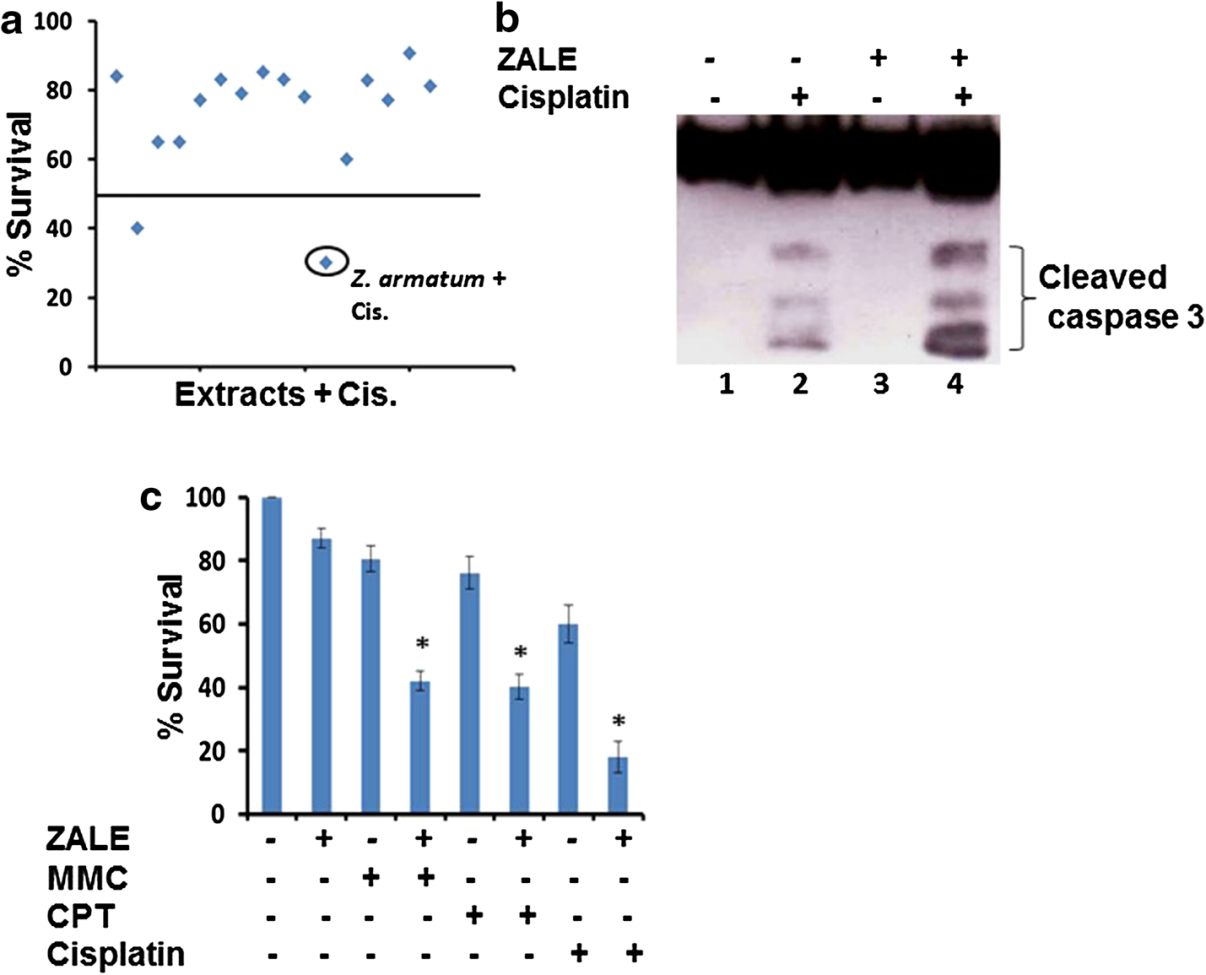
*ZALE* enhanced chemotherapeutic drugs induced apoptosis. **a** Screening of medicinal plants that increased the cisplatin induced cytotoxicity. HeLa cells were pretreated with the plant extracts (15 μg/ml) for 16 h before treatment with cisplatin (3 μM) for 72 h. Viable cells were measured. **b** Immunoblot showing that ZALE pretreatment increased cisplatin.induced caspase activation HeLa cells were treated as denoted and the cells were lysed and immunoblotted with caspase 3 antibody. **c** Sequential treatment of ZALE with chemotherapeutic drugs show synergistic apoptosis. HeLa cells were pre-treated with low dose (15 μg/ml) of ZALE for 16 h and then treated with 100 ng/ml of mitomycin C (MMC) or 10 ng/ml of camptothecin (CPT) or cisplatin (3 μM) for 72 h. Viable cells were measured as described else where *asterisk* indicates Q value more than 1.15 according to Jin’s formula

To investigate if any of the plant extracts screened in Fig. 1a, can enhanced the apoptosis induced by the chemotherapeutic drugs, we pretreated HeLa cells with low dose (15 μg/ml) of the plant extracts for 16 h and then exposed to chemotherapeutic drug, cisplatin for 48 h. Cell proliferation assay show that two of the plant extracts exhibit greater cell death in combination treatment, compared to the individual treatment. One of them is ZALE which showed 70 % more cell killing (Fig. 6a). This data suggest that ZALE also augment chemotherapeutic drugs induced apoptosis.

Caspase activation appears to be a common pathway in apoptosis induced by chemotherapeutic drugs. Because ZALE augment chemotherapeutic drugs induced apoptosis, we sought to examine the mechanism of this interaction by measuring caspase 3 activation by their cleavage. As shown in Fig. 6b, while ZALE alone cannot activate caspase 3, combination of ZALE with cisplatin resulted in enhanced caspase 3 activation. This data suggest that ZALE enhanced the apoptosis-inducing potential of chemotherapeutic drugs by triggering caspase-3 activation. Similar enhanced apoptosis was observed with other chemotherapeutic drugs such as mitomycin C and camptothecin (Fig. 6c). The potential synergistic effects were investigated using low doses of ZALE and chemotherapeutic drugs. Analysis of the combination effects in HeLa cells showed that Q values of all were more than 1.15, as shown in Table 1. The results showed that the combination of ZALE and chemotherapeutic drugs had an apparent effect on HeLa cells survival, and they had a synergistic effect. Analysis of the combination effects of ZALE and cisplatin in different cancer cell lines show that the synergistic effect is cancer cell type dependent. The synergistic effect was observed in cervical (HeLa), prostate (LNCaP), bone (U2OS) cancer cell lines but not in lungs cancer (A549) cell line (Table 2).

**Table 1:** Synergistic effect of ZALE combined with different drugs on HeLa cells analyzed by Jin’s formula (value listed in the table)

Chemotherapeutic drugs	ZALE (15 ug/mL)
MMC	2.14
Cisplatin	1.85
CPT	1.85

According to Jin’s formula,  < 0.85 indicates antagonism, 0.85 ≤  < 1.15 indicates additive effects, and  ≥ 1.15 indicates synergism

**Table 2:** Table showing the consequences of combination treatment of ZALE and cisplatin in different tumor cell lines

Cell lines	Cancer types	Synergistic effect of ZALE and cisplatin
LNCaP	Prostate	+
HeLa	Cervical	+
A549	Lungs	–
U2OS	Bone (osteosarcoma)	+

+ Synergistic effect observed and − synergistic effect not observed

## Discussion

The present experiments were designed to screen 16 medicinal plants for cytotoxicity and enhancing the efficacy of chemotherapeutic drugs. Here, we show that the crude ZALE induced cytotoxicity and also enhanced the effect of chemotherapeutic drugs. Evaluation of DAPI stained nuclei of HeLa cells after treating with ZALE show DNA fragmentation and apoptotic bodies formation (Fig. 1c). The amount of apoptotic bodies formation was in dose dependent manner; suggesting that ZALE induced apoptosis in HeLa cells and the IC_50_ was 60 μg/ml. Apoptotic effect was further confirmed by DNA fragmentation assay on agarose gel electrophoresis and by single cell gel electrophoresis. Extracts from other *Zanthoxylum* species have shown to possess cytotoxic and genotoxic effects. *Zanthoxylum americanum* extract has shown cytotoxicity [19] and Rutaceline (a benzophenanthridine) isolated from *Zanthoxylummadagascarien*se has shown antiproliferative effect, alteration of cell cycle, apoptosis, and genotoxic damage in a human intestinal cancer cell line Caco-2 [20].

Our results also show that ZALE activated MAPK pathway and ERK activation was maximum as compared to p38 or JNK activation (Fig. 3). ERK activation was essential for ZALE induced apoptosis. Further, there was no activation of caspase 3 and PARP cleavage in the ZALE induced apoptosis in HeLa cells (Fig. 2). Such caspase and PARP activation independent apoptosis was reported in rat adrenal pheochromocytoma PC12 cells while treated with α-eleostearic acid which is a conjugated trienoic fatty acid that occurs in the seeds of plants such as *Vernicia* spp. Such apoptosis is associated with superoxide production and the prolonged ERK phosphorylation and are not associated with molecules such as Bax, Cytochrome c caspase 3 and PARP [18].

ERK is a key regulator of apoptosis and the apoptotic cell death induced by several drugs such as camptothecin and *N*-methyl-*N*′-nitro-*N*-nitrosoguanidine (MNNG) can be blocked by MEK1/2 inhibitors (U0126). ERK function in response to DNA damage and ERK activation was observed in response to cisplatin in ovarian cancer cells [21]. We did not observe any MAPK activation in early time point (8 h). However, strong MAPK activation was observed at later time (18 h), indicating that the phenomenon might be due to the intrinsic cellular changes such as DNA damage. Interestingly, ZALE induced γH2AX foci formation which indicates DNA double strand breaks (Fig. 5). Degree of ERK activation correlated with the intensity of DNA damage. Tumor suppressor protein, p53 and ERK activation cooperate with DNA damage-induced cell cycle arrest and apoptosis [22]. Speculatively, ZALE might contain phytoactive compound(s) which induced DNA double strand breaks that activated MAPK pathway. Nevertheless, γH2Ax foci formation is also an indication of early apoptosis. It may also be formed concurrently with the initiation of DNA double-stranded breaks resulting from the apoptotic endonuclease activation and is essential for apoptosis that induced DNA fragmentation [21]. Interestingly, ZALE also enhanced the apoptosis inducing potential of chemotherapeutic drugs and the increased apoptosis was associated with increased caspase activation. It is well known that p38 is required for chemotherapeutic drugs induced apoptosis [23]. Since ZALE also induced p38 activation, it is possible that the enhanced apoptotic potential of chemotherapeutic drugs may be due to the activation of p38 by the ZALE (Fig. 3, lane 3).

Synergistic effect of ZALE and cisplatin were observed in various cancer cell lines (cervical, prostate, osteosarcoma) except A549 which is a lungs cancer cell line. Interestingly, all the cell lines which have shown synergistic combination effects, harbour wild type p53 while the A549 which did not show combination effects have mutant p53, suggesting that p53 might be essential for the increased apoptosis observed in the combination treatment.

The crude extract used is not of single chemical entities. Plants produced complex mixtures of secondary metabolites consisting of alkaloids, polyphenols, or diverse terpenoids and the extract will be a mixture of all the secondary metabolites [24]. Therefore, the biological activities of ZALE may be results of each independent constituent or an additive effect.

**Fig. 7:**
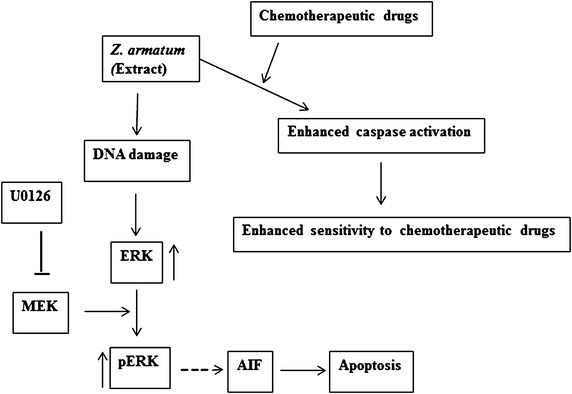
Speculative mechanism of ZALE mediated cell death. ZALE induced DNA damage and ERK activation. Activation of ERK released AIF that lead to apoptosis in a caspase independent manner. Alternatively, sequential treatment of ZALE with chemotherapeutic drugs enhanced caspase activation, leading to caspase dependent apoptosis

The properties of ZALE described here lead us to propose a model of its mechanism of action (summarized schematically in Fig. 7). Firstly, ZALE induced DNA double strand breaks as shown by increased γH2AX foci formation (Fig. 5) and the damaged DNA activated ERK pathway. Prolong activation of ERK leads to the release of apoptosis inducing factor (AIF) and thereby inducing apoptosis in a caspase independent pathway. Secondly, treatments of ZALE along with the chemotherapeutic drugs induced enhanced caspase activation that plays a significant role in the synergistic interaction among chemotherapeutic drugs and ZALE. Purification, identification and characterization of the bioactive compounds present in ZALE or administration of ZALE along with the chemotherapeutic drugs will open a new therapeutic window for cancer treatment.

## Conclusion

In conclusion, we have shown that crude ZALE induced apoptosis in human cervical cell line in an ERK dependent pathway; independent of caspase activation and PARP cleavage. ZALE also induced DNA damage and enhanced the chemotherapeutic drugs induced apoptosis by increasing caspase activation. We have shown the potential of combining chemotherapeutic drugs with ZALE for sensitizing the human cervical cancer cells to chemotherapeutic drugs.

## Methods

### Preparation of leaf extract

The study included 16 different plants from Manipur State of India and the leaf extracts were prepared following the protocol described earlier by Nostro et al. [24]. Briefly, the leaves were washed with pure water and dried at room temperature. The dried leaves were processed as coarse powder. Powdered material (10 g) was dissolved in methanol (100 mL) and incubated at 25 ± 1 °C for 48 h with agitation. The organic extract was filtered with Whatman filter paper No.1, concentrated by freeze drying. The dried extracts were dissolved in DMSO at 0.1 g/ml concentration and stored frozen at −20 °C until analysis.

### Antibodies and inhibitors

The antibodies for ERK, phospho-ERK, JNK, phospho JNK, p38, phospho-p38, cleaved caspase-3 and cleaved PARP-1 were purchased from Cell Signaling. β-actin (Santa Cruz Biotechnology) and γH2AX (Upstate) were also procured. The inhibitors, U0126 (Cell Signalling), SB203580 and SP600125 (Abcam) were also purchased. Working concentration for U0126, SB203580 and SP600125 are 10, 20 and 20 μM respectively.

### Chemicals

All the chemicals were purchased from Sigma Aldrich (USA). Mitomycin C (MMC) was dissolved in 70 % ethanol to a stock concentration of 250 ng/μL. Camptothecin (CPT) was dissolved in DMSO to a stock concentration of 10 mM.

### Cell culture

In order to evaluate anti-cancer activities of organic extract of *Zanthoxylum armatum.* DC, we have used different kinds of cancer cells. Within these cells we have included LNCaP, U2OS, A549 and HeLa. The LNCaP cells are human prostate cancer cells, highly resistant to human fibroblast interferon, and show an aneuploid (modal number, 76 to 91) human male karyotype with several marker chromosomes [25]. The U2OS cells are osteosarcoma epithelial cells with highly altered, chromosome counts in the hypertriploid range involving the same chromosomes (N1, N7, N9, and N11 particularly). The A549 cells are adenocarcinomic human alveolar basal epithelial cells. This is a hypotriploid human cell line with the modal chromosome number of 66, 64, 65, and 67 chromosome counts at relatively high frequencies. Finally the Hela cells are cells derived from cervical cancer cells with 100 % aneuploidy. There is a small telocentric chromosome in 98 % of the cells [26].

All the cells were purchased from the National Centre for Cell Science (Pune, India). The cells were maintained at 37 °C, 5 % CO2 in RPMI-1640 media supplement with 10 % fetal bovine serum (FBS).

### Evaluation of cell viability and apoptosis assay

HeLa cells were seeded in 96-well tissue culture plates, at a density of 5 × 10^3^ cells/well and incubated for 16 h to adhere the cells. The cells were then exposed to increasing concentrations of ZALE (15–90 μg/ml) for 48 h. and viable cells were assayed using CellTiter 96 AQueous One Solution Reagent (Promega) following the protocol provided by the manufacturer. For combination treatment, cells were pre-treated with the plant extracts for 16 h prior to the treatment with chemotherapeutic drugs.

For apoptosis assay, the cells were stained with 4, 6-diamidino-2-phenylindole (DAPI) and apoptotic cells are indicated by small, condensed nuclei. The cells were plated onto sterile coverslips in a 35 mm dish. After treatment, the floating cells were pelleted by centrifuging at 1100 rpm for 5 min. The cells were washed with PBS for 5 min. After aspirating the wash, the cells were permeabilized with 1 ml permeabilization buffer (0.5 % tritonX100 in PBS) for 3 min. The cells were washed and fixed in 1 ml of 2 % paraformaldehyde in PBS Buffer for 10 min. The cells were washed and mounted the coverslips on slides with mounting medium, ProLong Gold Antifade Reagent with DAPI (Invitrogen). The coverslip were glued using clear nail polish. Apoptotic cells can be observed with a fluorescent microscope.

### Evaluation of synergetic effect by Jin’s formula

Synergetic effect of the combination of ZALE and chemotherapeutic drugs was analyzed by Jin’s formula. The formula is* Q* =  *Ea* + *b*/(*Ea* + *Eb* – *Ea* × *Eb*), where *Ea* + *b*, represented the average effects (inhibition rate) of the combination treatment. and are the inhibition rate of single treatment of ZALE only or chemotherapeutic drug only. In this method,  < 0.85 indicates antagonism, 0.85 ≤ * Q* < 1.15 indicates additive effects, and  * Q* ≥ 1.15 indicates synergism.

### Immunofluorescent staining

For detection of γH2AX, cells were fixed with 4 % paraformaldehyde for 10 min. Cells were then permeabilized with PBS containing 0.5 % Triton X-100 for 5 min at RT. γH2AX foci were determined by immunofluorescence microscopy as described previously using Ser139 rabbit polyclonal antibodies (Upstate) [27].

### Western blot analysis

The cells were treated with the extract and the cells were lysed directly with 2X Laemmli sample buffer and the samples were resolved by SDS-PAGE on 5–12 % gradient gels and transferred onto PVDF membranes. The membranes were blocked with 5 % non-fat milk for 1 h and incubated with primary antibodies (1:2000 dilution) at 4 °C overnight and then with secondary antibody (1:6000 dilution) for 1 h at room temperature. The blots were detected using chemiluminescent ECL system (GE Healthcare).

## Additional file

Additional file 1 ZALE induced apoptosis in HeLA cells
