# Supplementary material for: Anticancer properties and enhancement of therapeutic potential of cisplatin by leaf extract of Zanthoxylum armatum DC
Source: Biol Res. 2015 Aug 20;48(1):46. doi: 10.1186/s40659-015-0037-4 (PMC4545984; doi:10.1186/s40659-015-0037-4)
Supplement: Additional file 1 — ZALE induced apoptosis in HeLA cells [file 40659_2015_37_MOESM1_ESM.pdf]

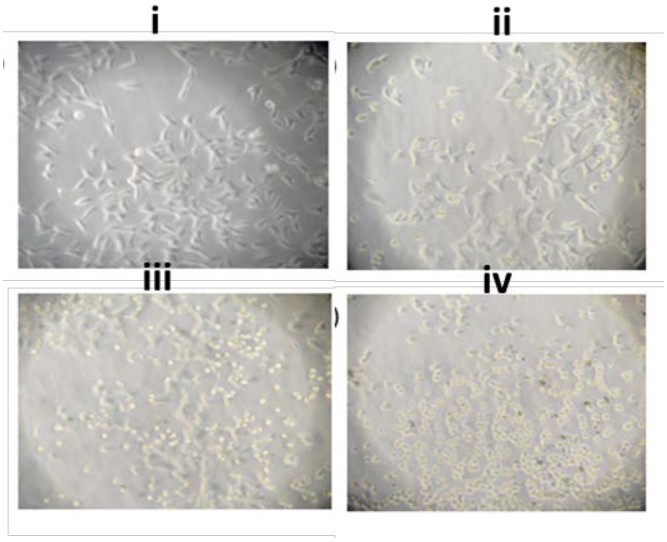

Suplementary Fig. S1. ZALE *is cytotoxic to HeLa cells.* Image showing cells treated with (i) DMSO, (ii) 30ug/mL, (iii) 60ug/mL (iv) 90ug/mL of the *Z. armatum* crude extract for 48 h.

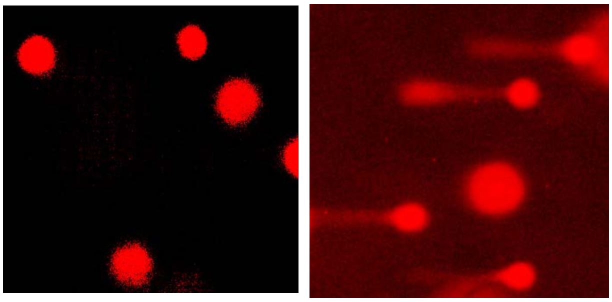

Suplementary Fig. S2. *HeLa cells treated with ZALE induced apoptosis.*. A. Representative image of Comet Assay for analyzing apoptotic cells of HeLa cells were treated with DMSO or 60ug/mL of ZALE for 24 h. Cells were lysed in agorose gel and electrophoresis under low current, the genomic DNA were stained with propidium iodide and observed under fluorescence microscope (Leica).
